# Osteoporosis risk in alpha and beta thalassemia: An age- and sex-specific retrospective cohort study

**DOI:** 10.1007/s11657-026-01711-y

**Published:** 2026-05-22

**Authors:** Yuan-Sheng Hsu, Sheng-Chieh Tseng, Tze-Fan Chao, Kun-Hui Chen

**Affiliations:** 1 Department of Orthopedic Surgery, Taichung Veterans General Hospital, 1650, Sec. 4, Taiwan Blvd., Xitun Dist., Taichung City, 407219 Taiwan; 2 PhD Program in Translational Medicine, National Chung Hsing University, Taichung, Taiwan; 3 Division of Cardiology, Department of Medicine, Taipei Veterans General Hospital, Taipei, Taiwan; 4 Institute of Clinical Medicine, and Cardiovascular Research Center, National Yang-Ming Chiao Tung University, Taipei, Taiwan; 5 Department of Post-Baccalaureate Medicine, College of Medicine, National Chung Hsing University, Taichung, 40227 Taiwan; 6 Department of Computer Science & Information Engineering, College of Computing and Informatics, Providence University, Taichung, Taiwan

**Keywords:** Thalassemia, Beta thalassemia, Osteoporosis, Thalassemia-associated osteoporosis, Real-world data

## Abstract

***Summary*:**

*Rationale:* Differential skeletal risks between alpha- and beta-thalassemia subtypes remain unclear. *Main result:* Beta-thalassemia correlates with higher fracture risk than alpha-thalassemia. Notably, males aged 18–50 with beta-thalassemia show a 14-fold increase in osteoporosis. *Significance:* Identifies a notably elevated osteoporosis risk in young males, supporting consideration of earlier skeletal surveillance.

**Purpose:**

The differences in osteoporosis and fracture risk between alpha- and beta-thalassemia subtypes, and how these differences vary by age and sex, remain poorly understood. We conducted a large-scale analysis to characterize these risks and guide risk-stratified protocols.

**Methods:**

This retrospective cohort study used the TriNetX health research network. Patients with alpha- and beta-thalassemia (identified by ICD-10 codes) were matched 1:1 with non-anemic controls by age, sex, race, and comorbidities (chronic kidney disease, diabetes mellitus, rheumatoid arthritis, malnutrition, and endocrine dysfunction) using propensity scores. We assessed the risk of osteoporosis, fractures, and mortality from Kaplan–Meier analyses.

**Results:**

Beta-thalassemia patients have a significantly greater risk of osteoporosis (HR = 1.26, 95% CI: 1.11–1.43) than alpha-thalassemia patients. Within the beta-thalassemia group, males demonstrated a risk of osteoporosis that was more than three times higher than that of controls (HR = 3.28, 95% CI: 2.51–4.28), which was notably higher than that observed in females (HR = 1.42, 95% CI: 1.26–1.60). Young males (aged 18–50) demonstrated a markedly elevated risk of osteoporosis (HR = 13.97, 95% CI: 7.30–26.73) and mortality (HR = 6.31, 95% CI: 3.62–11.01) compared to matched controls.

**Conclusion:**

Beta-thalassemia is associated with worse skeletal outcomes than alpha-thalassemia. Young male patients demonstrate substantially elevated risk of osteoporosis and mortality. These findings highlight the need for age- and sex-specific risk assessment in thalassemia patients.

**Supplementary Information:**

The online version contains supplementary material available at 10.1007/s11657-026-01711-y.

## Introduction

Hemoglobinopathies impact around 300,000–500,000 newborns a year, with beta-thalassemia and sickle cell disease the most common globally [1]. Thalassemia is characterized by defects in hemoglobin (Hb) synthesis, and this genetic defect hinders efficient erythropoiesis, leading to chronic anemia ranging from asymptomatic to severe conditions [2]. Beyond anemia, patients with thalassemia experience multiple chronic complications affecting quality of life, including cardiovascular dysfunction, endocrine disorders (particularly hypogonadism), and metabolic disturbances such as glucose intolerance [3].

Among these complications, thalassemia-associated osteoporosis (TAO) and increased fracture risk are particularly concerning [4]. A large-scale study utilizing the Thalassemia Clinical Research Network (TCRN) database reported an overall fracture prevalence of 12.1% among 702 thalassemia patients, including those with thalassemia major and other transfusion-dependent forms [5]. Beyond transfusion-dependent forms, beta-thalassemia intermedia also demonstrates elevated fracture risk. A nationwide, population-based cohort study revealed that even transfusion-naïve thalassemia patients have a 1.35-fold increased risk of fractures compared to non-thalassemia controls [6]. Sex-based differences are notable, with males experiencing higher osteoporosis risk and more severe bone disease than females [7]. A 19-year retrospective longitudinal study in 277 transfusion-dependent thalassemia patients found males have a higher osteoporosis incidence (16.5%) than females (7.7%) [8].

The two major forms, alpha- and beta-thalassemia, result from defects in alpha- and beta-globin chain synthesis, respectively. Despite growing recognition of TAO as a major complication, comparative data on skeletal risk between alpha- and beta-thalassemia subtypes are lacking. Furthermore, the age- and sex-specific risk remains poorly characterized.

Using real-world data from the global TriNetX health research network, we compared osteoporosis, fracture, and all-cause mortality risks between alpha- and beta-thalassemia patients. Age- and sex-stratified analyses were performed to characterize subtype-specific risk patterns and inform targeted screening strategies.

## Methods

### Data source and study design

This retrospective observational cohort study utilized de-identified patient data from the TriNetX Global Collaborative Network, which comprises electronic health records from healthcare organizations worldwide [9]. Cohorts were defined using specific ICD-10-CM codes, and clinical outcomes were compared between groups. Patient data were collected from January 1, 2015 onwards. The study population was restricted to adults aged 18 years and older at their index event. The data used in this study was collected on February 16, 2026 from the TriNetX Global Collaborative Network, which provided access to electronic medical records (diagnoses, procedures, medications, laboratory values, genomic information) from approximately 256,784 patients across 170 healthcare organizations.

### Case definition

The thalassemia cohort was identified through systematic application of International Classification of Diseases, Tenth Revision, Clinical Modification (ICD-10-CM) diagnostic codes, D56.0 for alpha-thalassemia and D56.1 for beta-thalassemia. From an initial pool of 187,314 adult patients with thalassemia, the final study cohorts comprised 28,128 individuals with alpha-thalassemia and 31,415 patients with beta-thalassemia.

### Control population selection

The control cohort was derived from individuals undergoing routine general medical examinations (ICD-10-CM code Z00.0). To ensure adequate healthcare engagement and minimize detection bias, controls were restricted to adults (age ≥ 18 years) with ≥ 10 documented clinical encounters from January 1, 2015 onwards. Patients with documented thalassemia or any form of anemia (ICD-10-CM codes D50-D64) were excluded to establish a comparison population free from related hematologic disorders. The final control cohort comprised 3,376,312 patients.

### Subgroup and negative control outcome analyses

Subgroup analyses were stratified by age (≤ 50 vs > 50 years), sex, and race/ethnicity to identify high-risk populations. Negative control outcome analysis was conducted using conditions unrelated to thalassemia pathophysiology (colonoscopy, appendicitis, acute upper airway infections, accidents, abrasions/contusions, and burns) to assess for residual confounding and differential surveillance. Additionally, consistency of findings across healthcare settings was evaluated through stratification by individual healthcare organizations.

### Outcome definitions

Primary outcomes were defined using ICD-10-CM codes. Osteoporosis was identified by codes M80 (osteoporosis with current pathological fracture) and M81 (osteoporosis without current pathological fracture). Fracture outcomes included: vertebral fractures (codes S22.0-S22.1 for thoracic vertebrae, S32.0 for lumbar vertebra, and S32.1 for sacrum), femoral fractures (code S72), and limb fractures (codes S42 for shoulder and upper arm, S52 for forearm, S62 for wrist and hand, S82 for lower leg, and S92 for foot and toe excluding ankle). All-cause mortality was determined by the presence of a "Deceased" designation in the patient record. Outcomes were assessed from the index date (first thalassemia diagnosis or health examination) through the end of available follow-up.

### Kaplan–meier survival analysis

Time-to-event analyses were performed using Kaplan–Meier methods to estimate cumulative incidence of outcomes. Patients with prevalent outcomes (present before the index date) were excluded. Follow-up extended from the index date to either the outcome occurrence or last recorded clinical encounter (censoring). Survival curves were compared using the log-rank test, and hazard ratios (HRs) with 95% confidence intervals (CIs) were calculated to quantify risk differences between groups.

### Propensity score matching

Propensity score matching (PSM) was performed to balance baseline characteristics and minimize confounding. The propensity score estimated the probability of having thalassemia based on demographic factors (age at index, sex, race/ethnicity) and clinical comorbidities (chronic kidney disease, diabetes mellitus, rheumatoid arthritis, malnutrition, and endocrine dysfunction including hypogonadism). Patients were matched 1:1 using nearest-neighbor matching without replacement, within a caliper width of 0.1 of the pooled standard deviation of the logit of the propensity score. Covariate balance was assessed using standardized mean differences (SMD), with SMD < 0.1 indicating adequate balance. All outcome analyses were performed on propensity-matched cohorts.

### Statistical analysis

Baseline characteristics were summarized using means with standard deviations for continuous variables and frequencies with percentages for categorical variables. Covariate balance after propensity score matching was assessed using standardized mean differences (SMD < 0.1). Subgroup analyses were stratified by age (≤ 50 vs > 50 years), sex, and race/ethnicity. A two-sided p-value < 0.05 was considered statistically significant. All analyses were conducted using the TriNetX Analytics platform.

### Missing data management

For demographic variables (age, sex, race/ethnicity), records with missing or unknown values were excluded from propensity score matching to ensure complete case analysis. Clinical comorbidities were defined as binary variables based on the presence of ICD-10-CM codes; absence of documented diagnosis was interpreted as absence of the condition. Missing outcome data were assumed to follow a missing-at-random (MAR) mechanism, as missingness was likely related to healthcare utilization patterns rather than outcomes themselves. Patients without follow-up visits after the index date were excluded from time-to-event analyses.

## Results

### Baseline characteristics

**Fig. 1:**
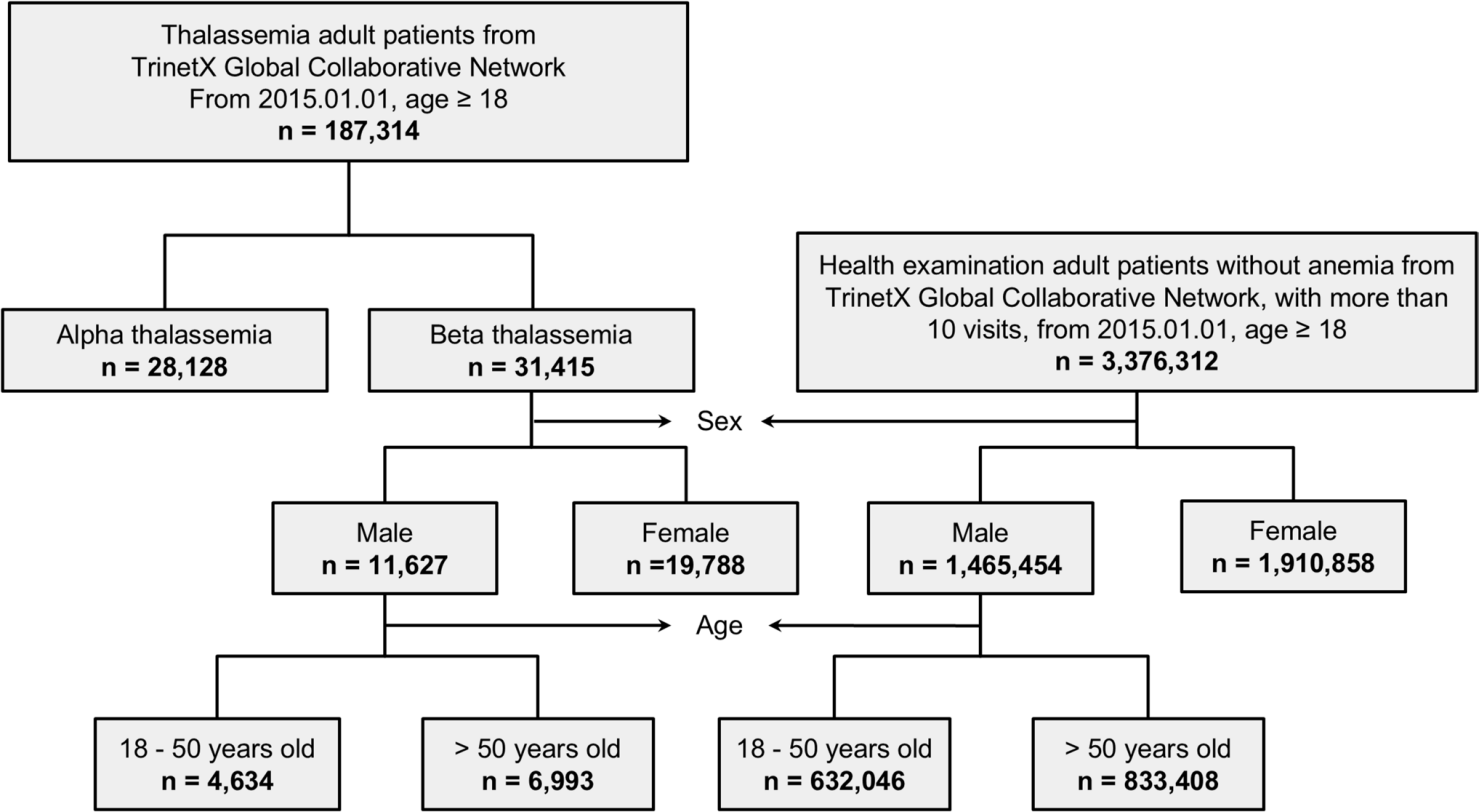
Patient flowchart showing selection and stratification of thalassemia patients and matched controls from the TriNetX Global Collaborative Network. Adult patients (age ≥ 18 years) with alpha-thalassemia (n = 28,128) and beta-thalassemia (n = 31,415) were identified from 2015 onwards. Control group consisted of patients receiving health examinations without anemia and with ≥ 10 clinical visits (n = 3,376,312). Stratification by sex and age groups (18–50 years vs > 50 years) shown for subsequent propensity score matching analyses

After propensity score matching, the study included 31,415 patients with beta-thalassemia matched 1:1 with controls, and 28,128 patients with alpha-thalassemia matched 1:1 with controls (Fig. 1). Baseline characteristics were well-balanced after matching, with all standardized mean differences < 0.1 (Table 1). Sex-stratified baseline characteristics are presented in Online Resource 1, and age-stratified characteristics for male patients are shown in Online Resource 2. Follow-up durations for all study cohorts and their respective age- and sex-stratified subgroups are detailed in Online Resource 3, including mean, standard deviation, and median values.

**Table 1:** Demographic of alpha and beta thalassemia before/after matching

	Alpha						Beta					
	Before Matching			After Matching			Before Matching			After Matching		
Char	Dis	Con	P (Std. Diff.)	Dis	Con	P (Std. Diff.)	Dis	Con	P (Std. Diff.)	Dis	Con	P (Std. Diff.)
Age	46.56 ± 20.36	48.35 ± 21.02	< 0.0001 0.0863	46.56 ± 20.36	46.63 ± 20.42	0.6979 0.0033	48.05 ± 20.36	48.35 ± 21.02	0.0127 0.0145	48.05 ± 20.36	48.09 ± 20.39	0.8329 0.0017
Male	9444 (34.353%)	1454725 (43.994%)	< 0.0001 0.1985	9444 (34.358%)	9407 (34.223%)	0.7396 0.0028	11349 (37.082%)	1454579 (43.982%)	< 0.0001 0.1409	11349 (37.083%)	11331 (37.025%)	0.8803 0.0012
Female	18047 (65.647%)	1851890 (56.006%)	< 0.0001 0.1985	18043 (65.642%)	18080 (65.777%)	0.7396 0.0028	19256 (62.918%)	1851764 (55.992%)	< 0.0001 0.1414	19255 (62.917%)	19269 (62.962%)	0.9067 0.0009
White	7766 (28.249%)	2407165 (72.798%)	< 0.0001 0.9953	7766 (28.253%)	7744 (28.173%)	0.8348 0.0018	12992 (42.451%)	2407519 (72.796%)	< 0.0001 0.6453	12992 (42.452%)	12978 (42.406%)	0.9088 0.0009
African American	8035 (29.228%)	324510 (9.814%)	< 0.0001 0.5052	8032 (29.221%)	8008 (29.134%)	0.8218 0.0019	6009 (19.634%)	324601 (9.815%)	< 0.0001 0.2798	6009 (19.635%)	5978 (19.533%)	0.7522 0.0026
Asian	3362 (12.229%)	136100 (4.116%)	< 0.0001 0.2995	3362 (12.231%)	3373 (12.271%)	0.8862 0.0012	3279 (10.714%)	136109 (4.116%)	< 0.0001 0.2539	3279 (10.714%)	3292 (10.757%)	0.8652 0.0014
Latino	17714 (64.436%)	2236143 (67.626%)	< 0.0001 0.0674	17710 (64.43%)	17685 (64.34%)	0.8238 0.0019	19981 (65.287%)	2236520 (67.626%)	< 0.0001 0.0496	19980 (65.286%)	19940 (65.155%)	0.7343 0.0027
Malnutrition	1297	16321	< 0.0001 0.2675	1293	1280	0.7929 0.0022	1405	16335	< 0.0001 0.2625	1404	1404	1.0000 0.0000
Diabetes mellitus	4808	370427	< 0.0001 0.1801	4804	4774	0.7359 0.0029	4843	370527	< 0.0001 0.1355	4843	4820	0.7987 0.0021
Kidney disease	4027	157722	< 0.0001 0.3384	4023	3982	0.6201 0.0042	4260	157761	< 0.0001 0.3183	4259	4214	0.5984 0.0043
Endocrine disorders	188	29,715	0.0002 0.0242	188	167	0.2635 0.0095	234	29,731	0.0131 0.0148	234	205	0.1648 0.0112
Rheumatoid arthritis	157	14673	0.0016 0.0179	156	150	0.7309 0.0029	175	14674	0.0008 0.0180	175	162	0.4776 0.0057

Abbreviations Dis, Disease; Con, Control; Std. Diff., Standardized Difference

### Differing impact of alpha- and beta-thalassemia on osteoporosis and fracture risk

**Fig. 2:**
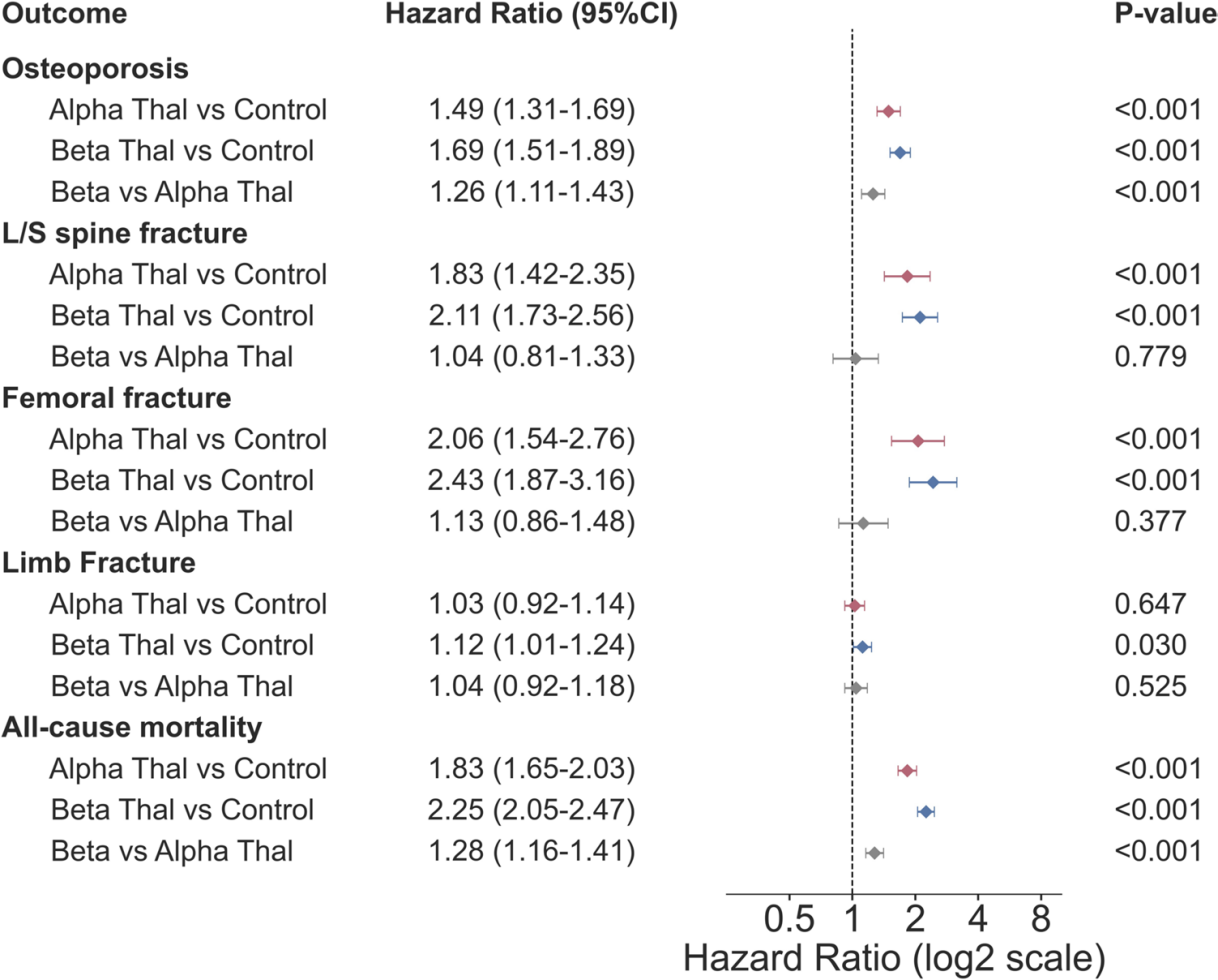
Forest plot comparing hazard ratios for skeletal and mortality outcomes in alpha-thalassemia and beta-thalassemia. Outcomes include osteoporosis, lumbar/sacral (L/S) spine fracture, femoral fracture, limb fracture, and all-cause mortality. Three comparisons shown for each outcome: alpha-thalassemia versus matched controls (red), beta-thalassemia versus matched controls (blue), and beta-thalassemia versus alpha-thalassemia (gray). Hazard ratios (HR) and 95% confidence intervals (CI) were estimated after propensity score matching. Hazard ratios displayed on log2 scale

First, we analyzed the risk of skeletal events and mortality among patients with alpha- and beta-thalassemia compared with controls, as well as between the two thalassemia groups (Fig. 2). Both thalassemia types demonstrated a significantly elevated risk profile compared to the control group. The hazard ratio for developing osteoporosis was higher in patients with beta-thalassemia (HR = 1.69, 95% CI: 1.51–1.89, p < 0.001), followed by alpha-thalassemia (HR = 1.49, 95% CI: 1.31–1.69, p < 0.001). A direct comparison confirmed that patients with beta-thalassemia carried a significantly higher risk than those with alpha-thalassemia (HR = 1.26, 95% CI: 1.11–1.43, p < 0.001).

**Fig. 3:**
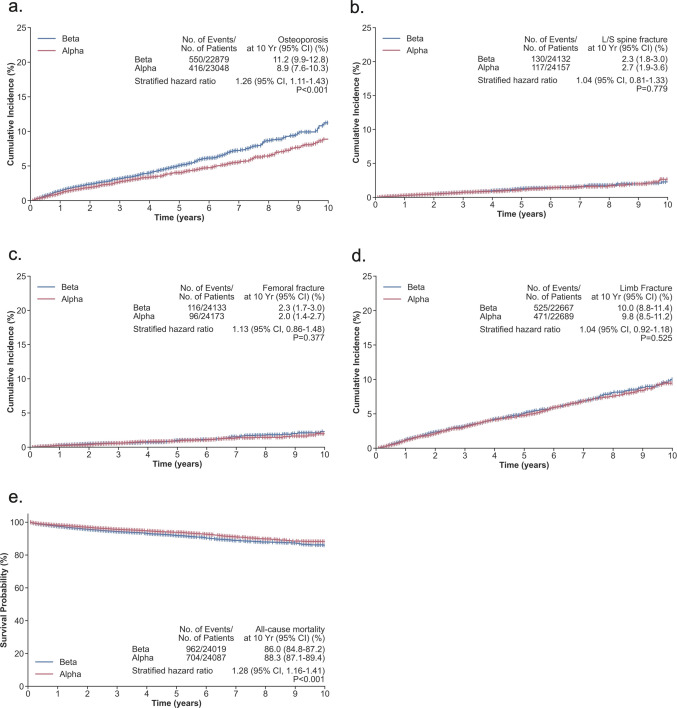
Kaplan–Meier survival curves comparing time-to-event outcomes between beta-thalassemia (blue) and alpha-thalassemia (red) patients. **a** Osteoporosis, **b** lumbar/sacral spine fracture, **c** femoral fracture, **d** limb fracture, **e** all-cause mortality. The 10-year event counts, cumulative incidence rates with 95% confidence intervals, hazard ratios, and log-rank p-values are shown in stat boxes

Regarding fracture risk, both groups showed substantial susceptibility. The risk of femoral fracture was elevated in both alpha-thalassemia (HR = 2.06, 95% CI: 1.54–2.76, p < 0.001) and beta-thalassemia (HR = 2.43, 95% CI: 1.87–3.16, p < 0.001), with no statistically significant difference between the two thalassemia types (HR = 1.13, 95% CI: 0.86–1.48, p = 0.377). Lumbar/sacral spine fracture risk was similarly elevated in both cohorts (beta-thalassemia: HR = 2.11, 95% CI: 1.73–2.56, p < 0.001; alpha-thalassemia: HR = 1.83, 95% CI: 1.42–2.35, p < 0.001), with no significant difference between groups (HR = 1.04, 95% CI: 0.81–1.33, p = 0.779). Limb fracture risk showed a modest elevation only in beta-thalassemia (HR = 1.12, 95% CI: 1.01–1.24, p = 0.030), while alpha-thalassemia showed no significant increase (HR = 1.03, 95% CI: 0.92–1.14, p = 0.647). All-cause mortality risk remained significantly higher in the beta-thalassemia group (HR = 2.25, 95% CI: 2.05–2.47, p < 0.001) compared to alpha-thalassemia (HR = 1.83, 95% CI: 1.65–2.03, p < 0.001), with a direct comparison hazard ratio of 1.28 (95% CI: 1.16–1.41, p < 0.001). Kaplan–Meier curves for all five outcomes comparing beta- and alpha-thalassemia are presented in Fig. 3.

To evaluate the consistency of our findings, we performed stratified analyses across racial groups and individual healthcare organizations (HCOs). The elevated risk of osteoporosis in beta-thalassemia remained consistent across all major racial subgroups, including White, Black or African American, Asian, and Hispanic or Latino populations (Online Resource 4). Furthermore, a meta-analytical approach across participating HCOs confirmed that the increased risk was not driven by specific center-level practices or geographic variations, demonstrating a uniform trend across the global federated network (Online Resource 5).

To address potential surveillance bias—the possibility that thalassemia patients appear to have more osteoporosis simply because they receive more frequent medical attention—we conducted a negative control outcome (NCO) analysis. We assessed the risk of several acute or traumatic events unrelated to thalassemia pathophysiology, such as colonoscopy, appendicitis, acute upper airway infections, accidents, abrasions/contusions, and burns. The hazard ratios for these NCOs were consistently near the null value (HR 1.0) with 95% confidence intervals crossing 1.0, suggesting that our primary findings are not significantly influenced by healthcare utilization bias or residual confounding (Online Resource 6).

### Sex difference of risk among beta-thalassemia patients

**Fig. 4:**
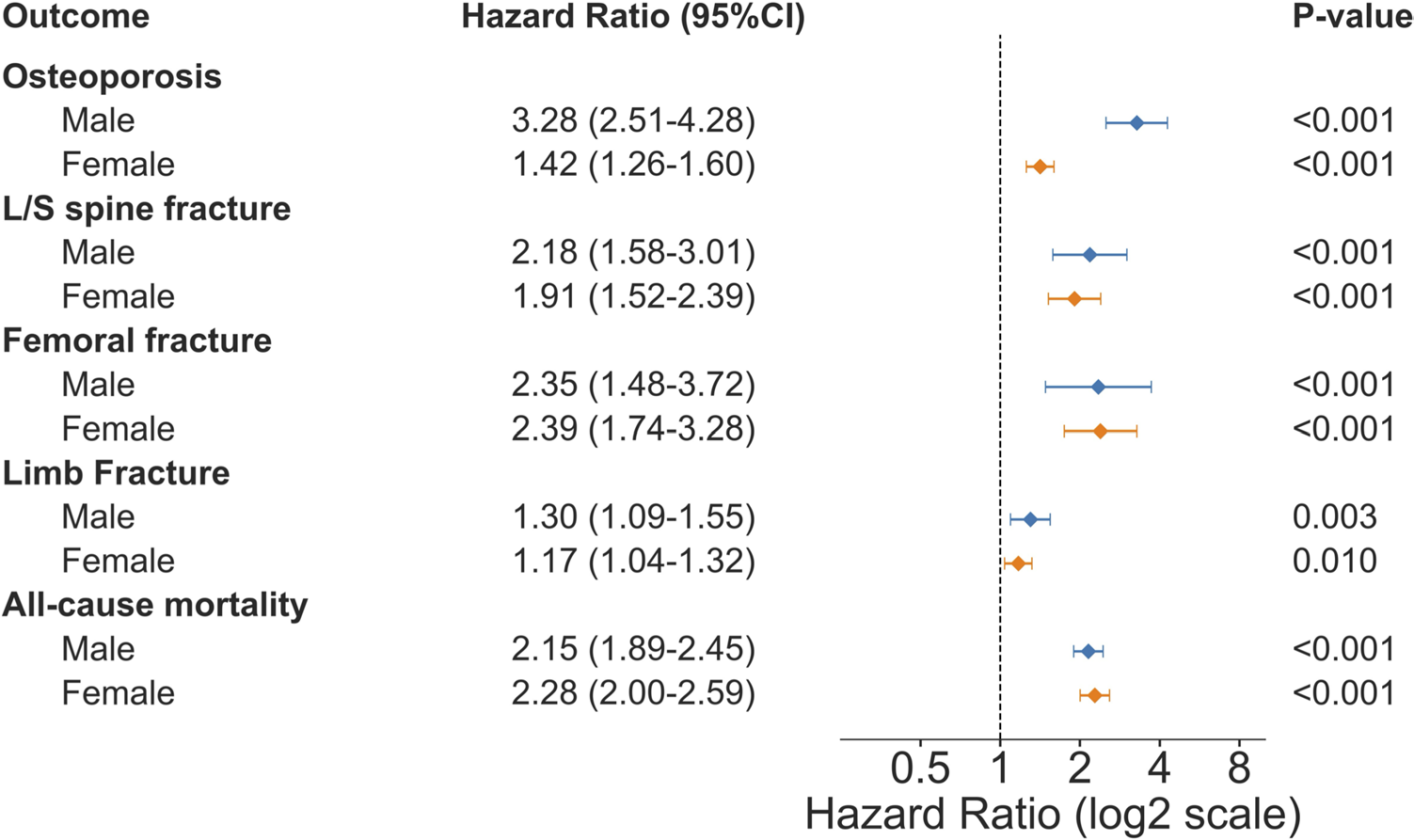
Sex-stratified forest plot comparing hazard ratios for skeletal and mortality outcomes in males (blue) versus females (orange) with beta-thalassemia compared to matched controls. Outcomes include osteoporosis, lumbar/sacral (L/S) spine fracture, femoral fracture, limb fracture, and all-cause mortality. Hazard ratios (HR) and 95% confidence intervals (CI) were estimated after propensity score matching. Hazard ratios displayed on log2 scale

Since earlier studies have reported that males with thalassemia tend to have more severe skeletal manifestations [7], we conducted sex-stratified analyses (Fig. 4) to validate and quantify these differences in our cohort. Both male and female patients demonstrated significantly elevated risks across all outcomes. However, sex-based divergence in osteoporosis risk was particularly substantial: male patients showed a 3.3-fold increased risk of osteoporosis (HR = 3.28, 95% CI: 2.51–4.28, p < 0.001) compared to a more moderate 42% increase in female patients (HR = 1.42, 95% CI: 1.26–1.60, p < 0.001). For fracture outcomes, male patients maintained higher hazard ratios for lumbar and sacral spine fractures (HR = 2.18, 95% CI: 1.58–3.01, p < 0.001) compared to females (HR = 1.91, 95% CI: 1.52–2.39, p < 0.001). Femoral fracture risk was substantially elevated in both sexes, with similar point estimates for females (HR = 2.39, 95% CI: 1.74–3.28, p < 0.001) and males (HR = 2.35, 95% CI: 1.48–3.72, p < 0.001). Limb fracture risk was modestly but significantly elevated in both males (HR = 1.30, 95% CI: 1.09–1.55, p = 0.003) and females (HR = 1.17, 95% CI: 1.04–1.32, p = 0.010). All-cause mortality risk was substantially elevated in both males (HR = 2.15, 95% CI: 1.89–2.45, p < 0.001) and females (HR = 2.28, 95% CI: 2.00–2.59, p < 0.001).

### Age-stratified analysis reveals severe early-onset osteoporosis in young males

**Fig. 5:**
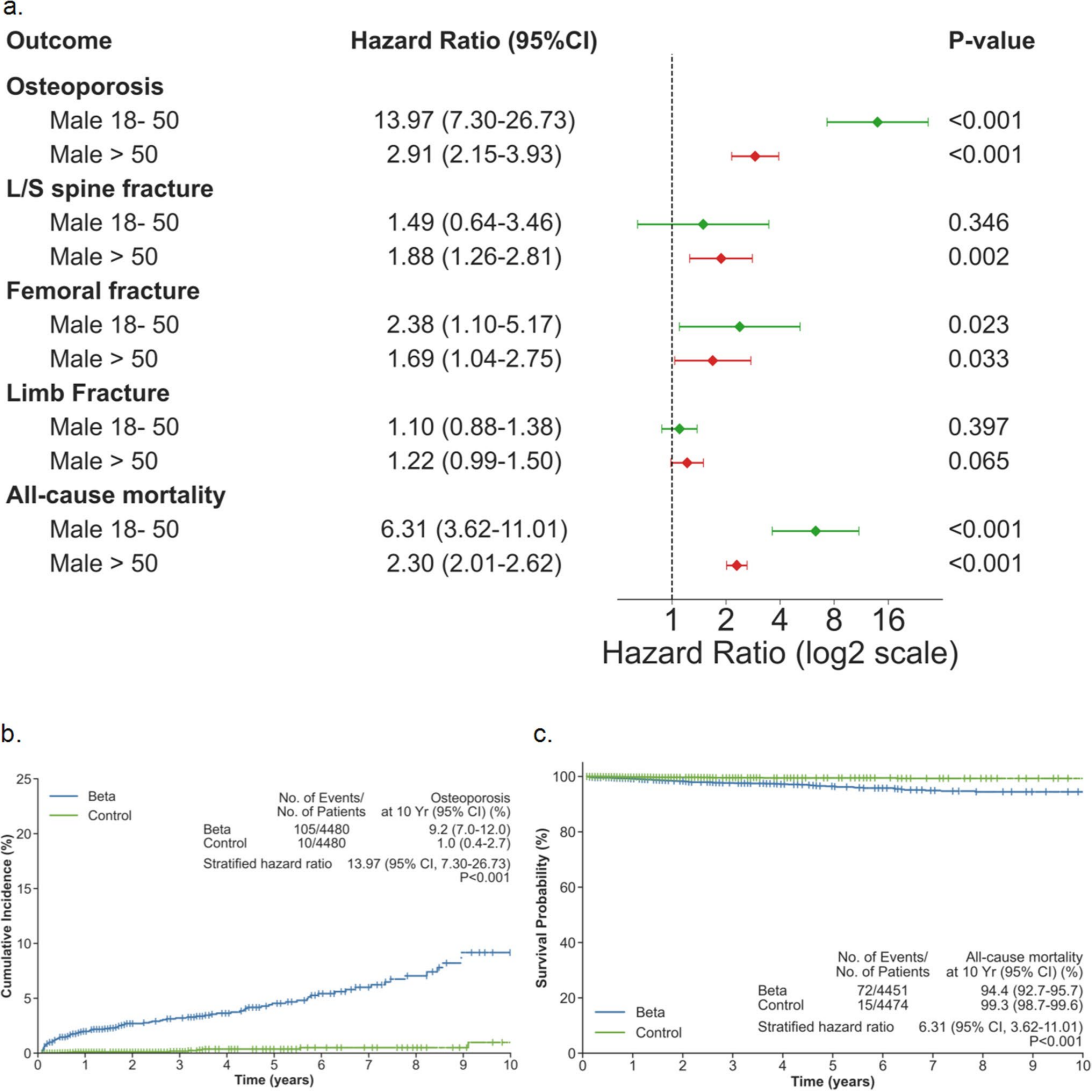
Age-stratified analysis of skeletal and mortality outcomes in males with beta-thalassemia compared to matched controls. **a** Forest plot comparing hazard ratios in young males (18–50 years, green) versus older males (> 50 years, red). Outcomes include osteoporosis, lumbar/sacral (L/S) spine fracture, femoral fracture, limb fracture, and all-cause mortality. Hazard ratios (HR) and 95% confidence intervals (CI) were estimated after propensity score matching. Hazard ratios displayed on log2 scale. **b** Kaplan–Meier curve for osteoporosis in young males. **c** Kaplan–Meier curve for all-cause mortality in young males. The 10-year event counts, cumulative incidence rates with 95% confidence intervals, hazard ratios, and p-values are shown in stat boxes

Given the pronounced sex-based differences in osteoporosis risk, we further stratified male patients by age to examine whether younger males showed signs of early-onset disease (Fig. 5). Males aged 18–50 years exhibited markedly elevated risk profiles. Osteoporosis risk was 14-fold higher (HR = 13.97, 95% CI: 7.30–26.73, p < 0.001), with 10-year cumulative incidence of 9.2% (105 events among 4,480 patients) in beta-thalassemia compared to 1.0% (10 events among 4,480 patients) in controls. All-cause mortality risk was also substantially elevated (HR = 6.31, 95% CI: 3.62–11.01, p < 0.001).

While risks remained statistically significant among males over 50 years, effect sizes were markedly attenuated. Osteoporosis risk remained 2.9-fold elevated (HR = 2.91, 95% CI: 2.15–3.93, p < 0.001) and mortality risk 2.3-fold elevated (HR = 2.30, 95% CI: 2.01–2.62, p < 0.001) in older males. Vertebral fracture risk was not significantly elevated in younger males (HR = 1.49, 95% CI: 0.64–3.46, p = 0.346) but was significantly increased in older males (HR = 1.88, 95% CI: 1.26–2.81, p = 0.002). Limb fracture risk showed no significant elevation in either younger males (HR = 1.10, 95% CI: 0.88–1.38, p = 0.397) or older males (HR = 1.22, 95% CI: 0.99–1.50, p = 0.065). Femoral fracture risk was significantly elevated in both age groups, with higher hazard ratio in younger males (HR = 2.38, 95% CI: 1.10–5.17, p = 0.023) compared to older males (HR = 1.69, 95% CI: 1.04–2.75, p = 0.033).

## Discussion

### Principal findings

This large-scale study is the first report to compare the risk of osteoporosis and fractures among different thalassemia subtypes using a propensity-matched cohort adjusted for key comorbidities. We identified three notable associations. First, a markedly elevated early-onset osteoporosis risk was observed in young males (≤ 50 years) with beta-thalassemia, who showed a 14-fold greater risk of osteoporosis (HR = 13.97, 95% CI: 7.30–26.73) and sixfold higher mortality (HR = 6.31, 95% CI: 3.62–11.01) compared to matched controls. Second, beta-thalassemia was associated with significantly higher skeletal disease burden than alpha-thalassemia (HR = 1.26, 95% CI: 1.11–1.43). Third, a pronounced sex disparity was observed, with males exhibiting 3.3-fold increased osteoporosis risk (HR = 3.28, 95% CI: 2.51–4.28) versus 42% increase in females (HR = 1.42, 95% CI: 1.26–1.60).

These findings suggest that young males with beta-thalassemia may represent a high-risk population who could benefit from proactive skeletal surveillance and targeted interventions to preserve skeletal health and reduce mortality. However, these effect estimates should be interpreted with caution, as outcomes were ascertained through ICD coding without direct BMD confirmation, and differential screening practices in younger males may contribute to the magnitude of the observed hazard ratios.

### Sex-specific risk patterns and clinical implications

Our analysis reveals significant sex-based differences in the impact of beta-thalassemia on the skeleton. Males had substantially higher risk of osteoporosis (HR = 3.28, 95% CI: 2.51–4.28), suggesting that beta-thalassemia is associated with disrupted bone homeostasis through mechanisms beyond normal age-related bone loss. This disproportionate increase implies disease-specific effects on male skeletal metabolism, likely exacerbated by hypogonadism and iron toxicity.

In contrast, females showed a 42% increased risk (HR = 1.42, 95% CI: 1.26–1.60), indicating that beta-thalassemia appears to act additively on pre-existing hormonal and metabolic vulnerabilities. The lower hazard ratio in females likely reflects the interaction between disease-related factors and baseline postmenopausal bone loss patterns, where thalassemia compounds rather than dominates the osteoporotic process.

These findings indicate that while beta-thalassemia is associated with compromised bone microarchitecture and structural integrity universally, the clinical presentation is distinct between sexes, supporting the consideration of sex-stratified fracture prevention strategies.

### Pathophysiological mechanisms of thalassemia-related osteoporosis

The bone loss experienced by individuals with thalassemia is a multifaceted problem resulting from several destructive factors.

i) The bone marrow expansion erodes the inner trabecular bone and thins the outer cortex, creating an environment that suppresses osteoblasts [10].
ii) Patients suffer from systemic iron overload resulting from increased intestinal absorption and the repeated blood transfusions required for treatment. This excess iron is toxic to the skeleton, inhibiting osteoblasts while promoting osteoclasts, which resorb bone, resulting in a net loss of bone mass [11].
iii) Iron toxicity damages the endocrine system, frequently causing hypogonadotropic hypogonadism. The resulting deficiency in protective sex hormones, such as estrogen and testosterone, removes a critical brake on bone resorption, dramatically accelerating bone degradation [12].
iv) Nutritional deficiencies compromise skeletal integrity. Thalassemia patients often have deficits in micronutrients such as vitamins A, C, D, selenium, and zinc, which are essential for bone metabolism and osteoblast function. Deficiencies correlate positively with age and iron overload severity, creating a compounding effect that exacerbates bone loss [13].

### Management of thalassemia-associated osteoporosis

The management of thalassemia-associated osteoporosis (TAO) requires a comprehensive, lifelong approach addressing its multifactorial pathogenesis. Early DXA scanning enables timely intervention through personalized protocols [14]. Foundation therapy centers on maintaining hemoglobin above 9–10 g/dL through regular transfusions, coupled with rigorous iron chelation. Concurrent management of hypogonadism and nutritional deficiencies, particularly vitamin D, calcium, and zinc, is essential for preserving skeletal integrity [15].

Pharmacological management primarily relies on bisphosphonates, with zoledronic acid being the most potent option. It suppresses osteoclast activity through inhibition of farnesyl pyrophosphate synthase and induction of apoptosis, restoring bone remodeling balance [16]. However, long-term safety data in thalassemia patients remain limited, particularly about atypical hip fractures or osteonecrosis of the jaw resulting from the long-term use of these drugs, due to a reduction in bone remodeling [17].

Alternative therapies show variable promise. Denosumab demonstrates uncertain efficacy, with recent evidence indicating equivocal effects for BMD Z-scores at 12 months. While potentially reducing bone pain, fracture prevention data remain absent [18]. Teriparatide, an anabolic agent, demonstrated 19–22% lumbar spine BMD increases and no new fractures in 11 thalassemia patients over 24 months. However, 45% experienced adverse effects including musculoskeletal pain, suggesting higher adverse event rates than non-thalassemia populations [19]. Romosozumab remains unstudied [20]. This highlights the need for large-scale trials to evaluate new treatments, with the primary goal being to prevent fractures.

### Perspectives and future directions

This study provides new insights into thalassemia-associated osteoporosis, particularly in young male beta-thalassemia patients, emphasizing the need to translate epidemiological findings into more accurate clinical risk models. Achieving this goal requires prospective longitudinal cohorts to examine the impact of disease subtypes, transfusion and chelation regimens, and endocrine status on bone loss trajectories. Such research would enable advancement from generalized to personalized risk stratification. A significant therapeutic gap also exists in developing bone anabolic agents for this population. Addressing these issues will pave the way for the next generation of thalassemia care.

### Limitations of the study

This study has several important limitations. The retrospective design and use of real-world data introduce potential information bias and unmeasured confounding.

Despite our expanded propensity score matching incorporating key osteoporosis-related comorbidities (chronic kidney disease, diabetes mellitus, rheumatoid arthritis, malnutrition, and endocrine dysfunction), residual confounding from unmeasured factors—including variations in patient adherence, transfusion frequency, compliance with chelation therapy, and disease severity—may remain. The absence of randomization precludes causal inference. Reliance on diagnostic codes also limits access to detailed clinical parameters such as specific bone density measurements.

Furthermore, the TriNetX database likely underrepresents populations with the highest thalassemia burden. According to Global Burden of Disease data, East Asia shows the highest age-standardized incidence rate (7.35 per 100,000 persons) and Southeast Asia the highest mortality rate (0.37 per 100,000 persons) for thalassemia [21]. These high-burden regions, particularly Thailand and Guangxi province, may have limited representation in the TriNetX database due to healthcare infrastructure disparities and database coverage gaps. This geographic bias substantially limits generalizability to populations where thalassemia is most prevalent, as medical resources, treatment protocols, and disease phenotypes in these regions may differ significantly from the predominantly Western populations captured in our dataset.

Osteoporosis was identified solely through ICD-10 diagnostic codes (M80-M81) without bone mineral density measurements, which introduces potential ascertainment bias. ICD-based ascertainment exhibits differential sensitivity across age groups: young male controls are rarely screened per clinical guidelines, leading to under-detection and inflated hazard ratios, while older adults have more complete ascertainment in both groups, yielding conservative estimates. Validation studies show ICD osteoporosis diagnosis codes have moderate positive predictive values (44–85%) but variable sensitivity (23–80%) [22], while fracture outcomes demonstrate higher positive predictive values (86–100%) [23]. To mitigate this bias, we required ≥ 10 clinical encounters for all controls and conducted negative control outcome analysis (Online Resource 6), which showed no significant associations with unrelated conditions.

Controls were derived from individuals receiving general health examinations, which may introduce healthy screenee bias and potentially inflate hazard ratios. However, this selection strategy was necessary to ensure comparable opportunity for outcome ascertainment between groups, as thalassemia patients receive regular specialty care with systematic monitoring. This methodological trade-off prioritizes minimizing detection bias over potential healthy screenee effects. Importantly, despite these mitigation strategies, detection bias is unlikely to be fully resolved. In particular, young male patients without thalassemia are rarely screened for osteoporosis under current clinical guidelines, whereas young males with thalassemia may undergo bone density assessment as part of routine disease monitoring. This differential screening intensity may inflate the observed hazard ratios in the younger male subgroup. Accordingly, the markedly elevated effect sizes in young males (HR = 13.97) should be interpreted as reflecting both a genuine biological risk and potential ascertainment asymmetry, and these subgroup findings warrant cautious interpretation pending prospective validation with BMD-confirmed endpoints.

Despite these limitations, this study represents the first large-scale comprehensive comparison of osteoporosis and fracture risk across thalassemia subtypes, leveraging substantial statistical power to identify important risk stratification patterns, particularly the markedly elevated osteoporosis risk observed in young males with beta-thalassemia that merits further clinical investigation.

Variations in clinical practice patterns across healthcare systems may further limit generalizability. These limitations underscore the importance of prospective validation studies incorporating underrepresented populations and comprehensive clinical parameters.

## Conclusion

This large-scale, multi-national study provides important insights into thalassemia-associated skeletal complications. Young males with beta-thalassemia showed a 14-fold increased risk of osteoporosis and a sixfold increase in mortality compared to matched controls. Beta-thalassemia was associated with a significantly greater risk of osteoporosis than alpha-thalassemia, although the risk of femoral fractures remains comparably elevated in both subtypes. A marked sex disparity was observed, with males showing a 3.3-fold increased risk of osteoporosis versus a 1.4-fold increase for females. These findings demonstrate that osteoporosis risk remains elevated across geographic regions and follow-up periods, supporting thalassemia-associated osteoporosis as a persistent, long-term complication. These findings support consideration of risk-stratified management approaches, particularly early, sex-specific bone health assessment in young males with beta-thalassemia and comprehensive fracture prevention strategies for all thalassemia patients. Validation in high-prevalence regions including East Asia, Southeast Asia, and the Mediterranean is essential before implementing risk-stratified screening protocols in these populations.

## Supplementary Information

Below is the link to the electronic supplementary material.

Supplementary file1 (DOCX 1488 KB)
