# Supplementary material for: Osteoporosis risk in alpha and beta thalassemia: An age- and sex-specific retrospective cohort study
Source: Arch Osteoporos. 2026 May 22;21(1):83. doi: 10.1007/s11657-026-01711-y (PMC13197373; doi:10.1007/s11657-026-01711-y)
Supplement: Supplementary file 1 — Supplementary file1 (DOCX 1488 KB) [file 11657_2026_1711_MOESM1_ESM.docx]

Electronic Supplementary Material

**Article Title:**

Osteoporosis Risk in Alpha and Beta Thalassemia: An Age- and Sex-Specific Retrospective Cohort Study

**Journal:**

Osteoporosis International

**Authors:**

Y.S. Hsu, S.C. Tseng, T.F. Chao, K.H. Chen

**Corresponding Author:**

K.H. Chen, Department of Orthopedic Surgery, 1650, Sec. 4, Taiwan Blvd., Xitun Dist., Taichung City 407219, Taiwan *Email* address:[Orthochen@gmail.com](mailto:Orthochen@gmail.com)

**Online Resource 1. Demographic of Male and Female Beta Thalassemia Before/After Matching**

|  | Beta M | | | | | | Beta F | | | | | |
| --- | --- | --- | --- | --- | --- | --- | --- | --- | --- | --- | --- | --- |
|  | Before Propensity Score Matching | | | After Propensity Score Matching | | | Before Propensity Score Matching | | | After Propensity Score Matching | | |
| Variant | Disease | Control | p(Std.Diff.) | Disease | Control | p(Std.Diff.) | Disease | Control | p(Std.Diff.) | Disease | Control | p(Std.Diff.) |
| Age | 52.09 ± 20.65 | 47.16 ± 21.41 | <0.0001 0.2342 | 52.08 ± 20.65 | 52.12 ± 20.63 | 0.89640.0018 | 45.70 ± 19.80 | 49.28 ± 20.66 | <0.0001 0.1769 | 45.71 ± 19.81 | 45.73 ± 19.82 | 0.92050.0010 |
| Male | 10959 (100%) | 1454579 (100%) |  | 10955 (100%) | 10955 (100%) |  | 0 (0%) | 0 (0%) |  | 0 (0%) | 0 (0%) |  |
| Female | 0 (0%) | 0 (0%) |  | 0 (0%) | 0 (0%) |  | 19258 (100%) | 1851866 (100%) |  | 19253 (100%) | 19253 (100%) |  |
| White | 5264 (48.034%) | 1059285 (72.824%) | <0.0001 0.5241 | 5264 (48.051%) | 5267 (48.079%) | 0.96760.0005 | 7573 (39.324%) | 1347771 (72.779%) | <0.0001 0.7159 | 7573 (39.334%) | 7567 (39.303%) | 0.95010.0006 |
| African American | 1739 (15.868%) | 139137 (9.565%) | <0.0001 0.1900 | 1737 (15.856%) | 1717 (15.673%) | 0.71080.0050 | 4223 (21.929%) | 185347 (10.009%) | <0.0001 0.3298 | 4221 (21.924%) | 4191 (21.768%) | 0.7114 0.0038 |
| Asian | 1005 (9.171%) | 58301 (4.008%) | <0.0001 0.2092 | 1005 (9.174%) | 1004 (9.165%) | 0.98130.0003 | 2200 (11.424%) | 77793 (4.201%) | <0.0001 0.2716 | 2199 (11.422%) | 2206 (11.458%) | 0.9108 0.0011 |
| Latino | 7226 (65.937%) | 982214 (67.526%) | 0.0004 0.0337 | 7222 (65.924%) | 7205 (65.769%) | 0.80860.0033 | 12396 (64.368%) | 1253812 (67.705%) | <0.0001 0.0705 | 12391 (64.359%) | 12363 (64.213%) | 0.76590.0030 |
| Malnutrition | 657 | 6307 | <0.0001 0.3193 | 653 | 656 | 0.93190.0012 | 719 | 10015 | <0.0001 0.2221 | 714 | 718 | 0.91420.0011 |
| Diabetes mellitus | 2220 | 182438 | <0.0001 0.2095 | 2218 | 2215 | 0.95980.0007 | 2565 | 187979 | <0.0001 0.0986 | 2563 | 2544 | 0.77530.0029 |
| Kidney disease | 2084 | 80173 | <0.0001 0.4207 | 2080 | 2057 | 0.69130.0054 | 2111 | 77546 | <0.0001 0.2582 | 2106 | 2083 | 0.70660.0038 |
| Endocrine disorders | 112 | 15363 | 0.7272 0.0034 | 112 | 93 | 0.18240.0180 | 118 | 14351 | 0.0106 0.0195 | 118 | 94 | 0.09840.0168 |
| Rheumatoid arthritis | 35 | 3332 | 0.0492 0.0173 | 35 | 30 | 0.53450.0084 | 138 | 11341 | 0.0655 0.0128 | 138 | 129 | 0.58050.0056 |

**Online Resource 2. Demographic of Male Beta Thalassemia young and old Before/After Matching**

|  | Beta M 18-50 | | | | | | Beta M >50 | | | | | |
| --- | --- | --- | --- | --- | --- | --- | --- | --- | --- | --- | --- | --- |
|  | Before Propensity Score Matching | | | After Propensity Score Matching | | | Before Propensity Score Matching | | | After Propensity Score Matching | | |
| Variant | Disease | Control | p(Std.Diff.) | Disease | Control | p(Std.Diff.) | Disease | Control | p(Std.Diff.) | Disease | Control | p(Std.Diff.) |
| Age | 30.21 ± 10.37 | 25.54 ± 12.86 | <0.0001 0.3997 | 30.19 ± 10.36 | 30.23 ± 10.36 | 0.8257 0.0047 | 66.28 ± 11.02 | 62.42 ± 10.13 | <0.0001 0.3647 | 66.27 ± 11.02 | 66.34 ± 11.04 | 0.7345 0.0058 |
| Male | 4487 (100%) | 601903 (100%) |  | 4480 (100%) | 4480 (100%) |  | 6863 (100%) | 852827 (100%) |  | 6856 (100%) | 6856 (100%) |  |
| Female | 0 (0%) | 0 (0%) |  | 0 (0%) | 0 (0%) |  | 0 (0%) | 0 (0%) |  | 0 (0%) | 0 (0%) |  |
| White | 1224 (27.279%) | 388616 (64.565%) | <0.0001 0.8068 | 1224 (27.321%) | 1216 (27.143%) | 0.8494 0.0040 | 4197 (61.154%) | 670753 (78.651%) | <0.0001 0.3886 | 4197 (61.216%) | 4208 (61.377%) | 0.8471 0.0033 |
| African American | 747 (16.648%) | 82078 (13.636%) | <0.0001 0.0841 | 743 (16.585%) | 742 (16.563%) | 0.97730.0006 | 1039 (15.139%) | 57085 (6.694%) | <0.0001 0.2733 | 1037 (15.125%) | 1028 (14.994%) | 0.82980.0037 |
| Asian | 512 (11.411%) | 28066 (4.663%) | <0.0001 0.2501 | 511 (11.406%) | 510 (11.384%) | 0.97350.0007 | 567 (8.262%) | 30242 (3.546%) | <0.0001 0.2011 | 563 (8.212%) | 564 (8.226%) | 0.97520.0005 |
| Latino | 2388 (53.22%) | 396714 (65.91%) | <0.0001 0.2608 | 2382 (53.17%) | 2373 (52.969%) | 0.84890.0040 | 5198 (75.739%) | 585590 (68.665%) | <0.0001 0.1584 | 5191 (75.715%) | 5195 (75.773%) | 0.93650.0014 |
| Malnutrition | 119 | 1829 | <0.0001 0.1955 | 112 | 103 | 0.53440.0131 | 567 | 4479 | <0.0001 0.3844 | 560 | 560 | 1.00000.0000 |
| Diabetes mellitus | 254 | 20069 | <0.0001 0.1124 | 251 | 242 | 0.67670.0088 | 2024 | 162375 | <0.0001 0.2456 | 2019 | 2014 | 0.92530.0016 |
| Kidney disease | 289 | 7344 | <0.0001 0.2746 | 283 | 273 | 0.66150.0093 | 1860 | 72831 | <0.0001 0.5000 | 1853 | 1849 | 0.93870.0013 |
| Endocrine disorders | 37 | 4504 | 0.5547 0.0086 | 37 | 34 | 0.72080.0076 | 79 | 10860 | 0.3679 0.0112 | 79 | 70 | 0.45850.0127 |
| Rheumatoid arthritis | 10 | 313 | <0.0001 0.0461 | 10 | 10 | 1.00000.0000 | 34 | 3019 | 0.0498 0.0217 | 34 | 29 | 0.52780.0108 |

**Online Resource 3. Follow-up Times of each cohort study**

|  | Cohort1 | | | | Cohort2 | | | |
| --- | --- | --- | --- | --- | --- | --- | --- | --- |
| Study | Name | Mean_Days | SD | Median_Days | Name | Mean_Days | SD | Median_Days |
| Alpha | Alpha | 778.23 | 1008.45 | 281 | Control | 1246.91 | 1079.99 | 948 |
| Beta | Beta | 837.21 | 1028.09 | 357 | Control | 1230.85 | 1075.83 | 922 |
| Beta.M | Beta.M | 732.82 | 978.02 | 253 | Control.M | 1203.42 | 1052.81 | 907 |
| Beta.F | Beta.F | 917.23 | 1048.49 | 456 | Control.F | 1263.17 | 1071.96 | 961 |
| Beta.old.M | Beta.old.M | 704.71 | 937.62 | 252 | Control.old.M | 1275.03 | 1051.63 | 992 |
| Beta.young. | Beta.young.M | 808.66 | 1050.05 | 299 | Control.young.M | 1037.16 | 992.11 | 732 |

**Online Resource 4. Racial Differences in Osteoporosis Risk Among Patients with
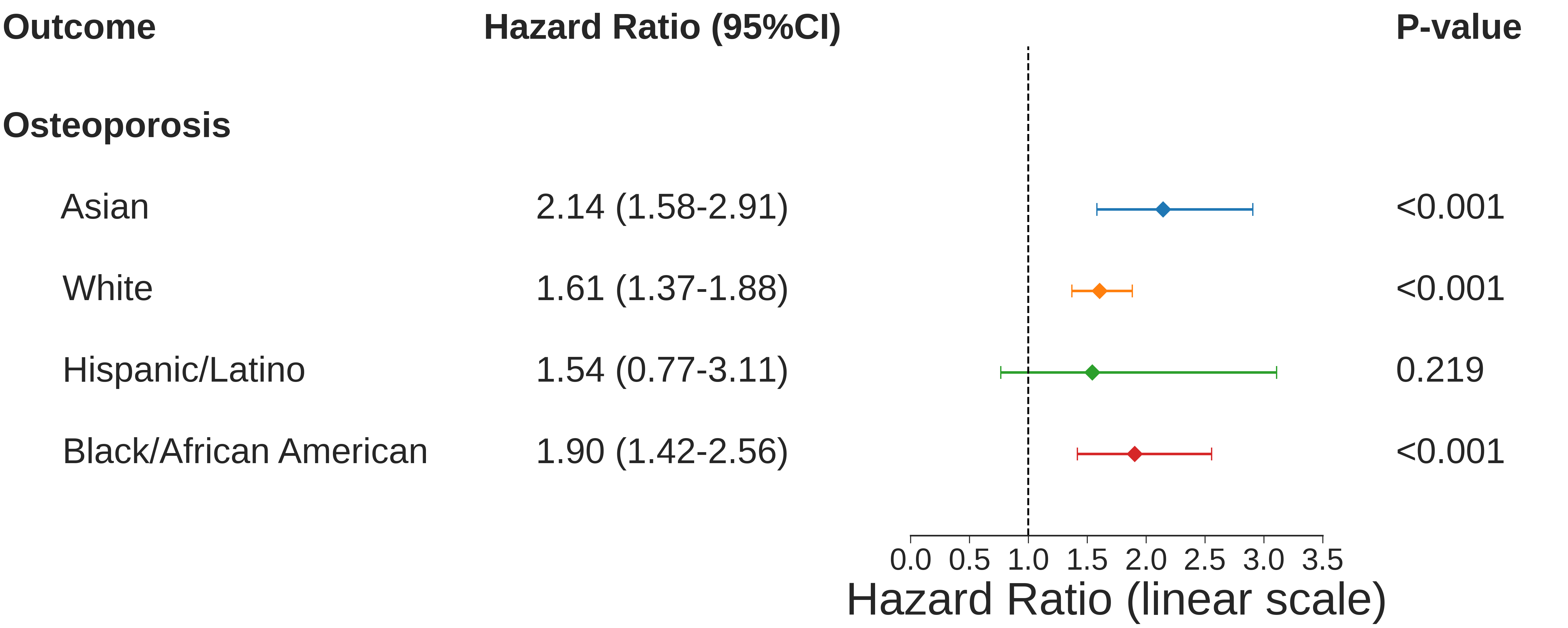
 Beta-Thalassemia.** Forest plot displaying the adjusted Hazard Ratios (HR) and 95% Confidence Intervals (CI) for the development of osteoporosis in patients with beta-thalassemia compared to matched non-anemic controls, stratified by race. The analysis evaluates risk magnitudes within Asian, White , Hispanic or Latino, and Black or African American subgroups. The vertical line at HR = 1.0 represents the null value (no difference in risk).

**Online Resource 5. Consistency of Osteoporosis Risk Across Participating
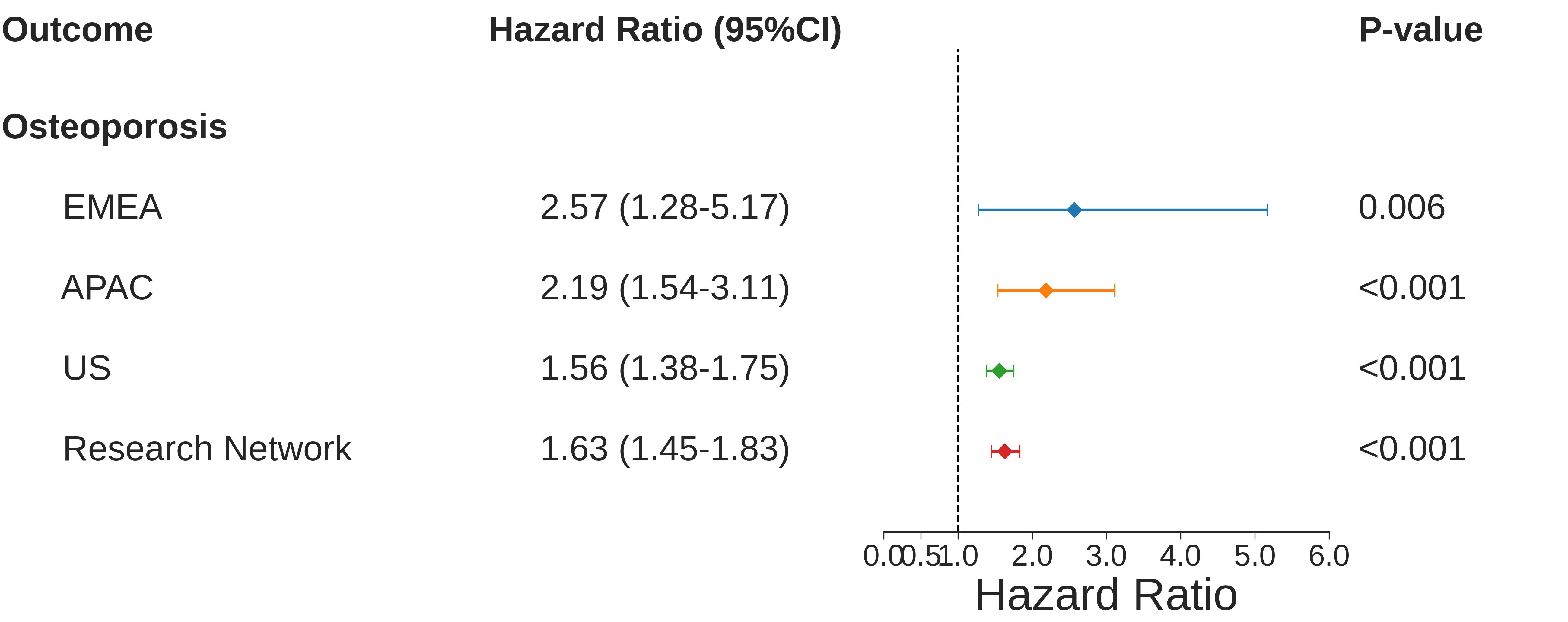
 Healthcare Organizations.** Forest plot illustrating the adjusted Hazard Ratios (HR) and 95% Confidence Intervals (CI) for osteoporosis in beta-thalassemia patients compared to matched controls, stratified by individual Healthcare Organizations (HCOs). Each square represents the point estimate for a specific HCO, with horizontal lines indicating the 95% CI. The vertical line at HR = 1.0 denotes no difference in risk.

**Online Resource 6. Evaluation of Negative Control Outcomes (NCO) to Assess
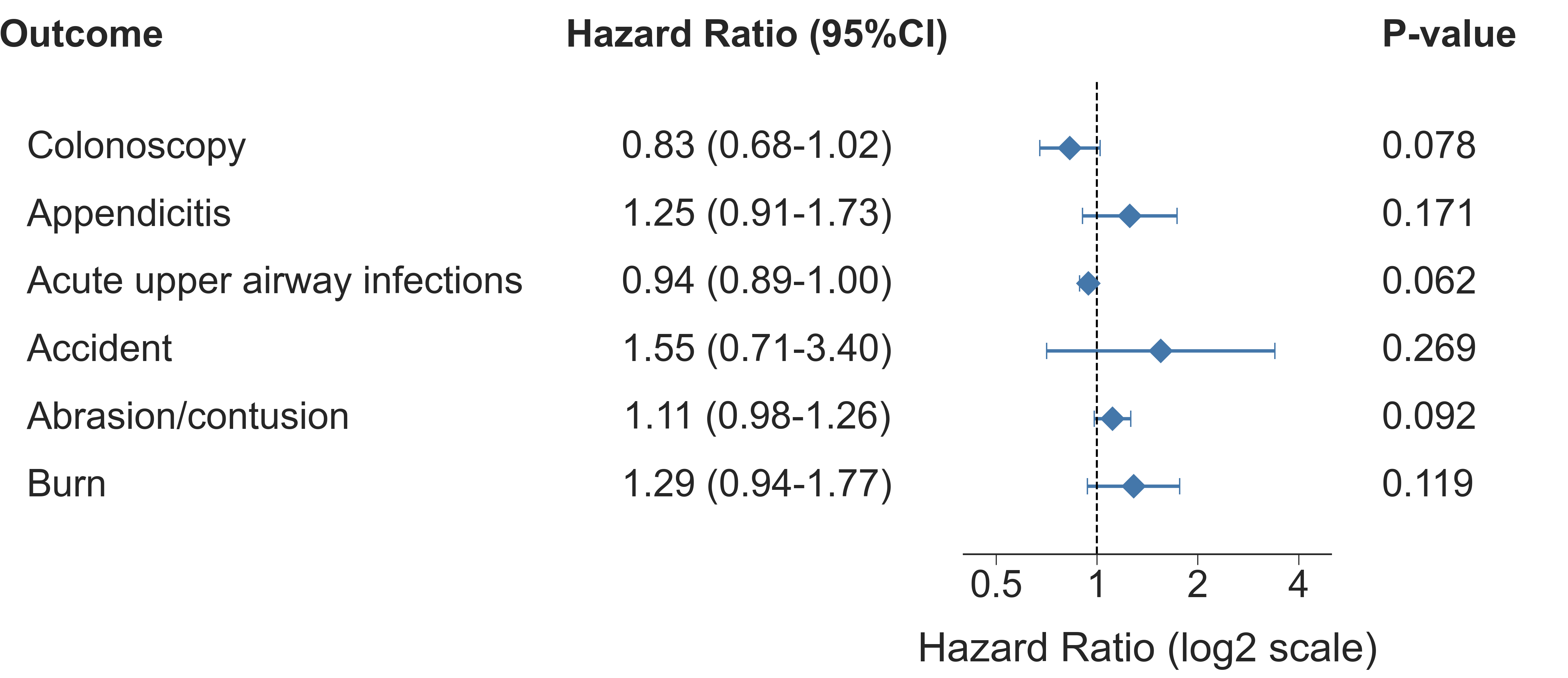
 Potential Bias.** (A) Forest plot displaying the adjusted Hazard Ratios (HR) and 95% Confidence Intervals (CI) for negative control outcomes in propensity-score-matched thalassemia patients compared to non-anemic controls. The analysis included a diverse set of acute, traumatic, and procedural outcomes unrelated to thalassemia pathophysiology: Colonoscopy, Appendicitis, Acute upper airway infections, Accidents, Abrasions/contusions, and Burns. The use of these negative controls helps to distinguish true disease-specific associations from potential surveillance bias or residual confounding related to healthcare utilization frequency. The x-axis uses a logarithmic scale to accommodate the range of risk estimates.

**Data Source and Privacy Compliance**

**A. Data Source**

TriNetX is a global health research network providing access to electronic medical records across large healthcare organizations. It provides data on demographics, diagnoses (ICD-10-CM), procedures (ICD-10 Procedure Coding System, Current Procedural Terminology, or Systematized Nomenclature of Medicine - Clinical Terms), prescriptions (Anatomical Therapeutic Chemical Classification system or RxNorm), laboratory tests (Logical Observation Identifiers Names and Codes), genetic data (Human Genome Variation Society standards), and healthcare service use. This report was run on a network of 83 HCOs. The analysis process includes two main steps: 1) Defining the cohorts through query criteria; 2) Setting up and running the analysis.

According to the detailed database description provided on the website: https://trinetx.com/trust-security/, TriNetX is recognized as an ISO/IEC 27001:2013 certified entity. This demonstrates its commitment to maintaining a robust Information Security Management System (ISMS). This international standard outlines the criteria for the creation, implementation, operation, surveillance, assessment, preservation and enhancement of a documented ISMS, and tailoring it to the specific risks faced by the organization. Emphasizing a risk-based methodology, ISO/IEC27001:2013 promotes suitable and balanced security measures to protect information assets. The ISMSunderpins the architecture and functionalities required to handle the data assets, personnel, operations and technology that form the backbone of the TriNetX platform. Further information regarding TriNetX's ISO/IEC 27001:2013 certification can be found in the Schellman ISO Certificate Directory (https://www.schellman.com/certificate-directory).

TriNetX has established systems and protocols that align with the European Union's (EU) criteria. extraterritorial transfer of data to its platform, emphasizing safeguarding and enhancing the confidentiality, integrity, availability and resilience of systems and services. To guarantee that information is adequately de-identified in accordance with the Health Insurance Portability and Accountability Act (HIPAA) Privacy Rule, TriNetX deploys the Expert Determination method. To this end, TriNetX utilizes the services of Bradley Malin, Ph.D utilized. Furthermore, TriNetX adheres to the standards set by the Brazilian General Data Protection Law (LGPD), demonstrating its commitment to transparency by entering into Data Protection addendums with Brazilian healthcare organizations. According to the agreements between HCOs and TriNetX, all data provided to TriNetX will undergo pseudonymization and/or de-identification by the HCOs. TriNetX also offers assistance to HCOs in establishing pseudonymization processes. If they are not already in place. Additionally, TriNetX uses expert determination to ensure that Data made available to end users is sufficiently de-identified. To manage data consistently, TriNetX has formed a Data Trust team comprising employees from its legal, privacy, compliance and security departments. This team is responsible for evaluating data throughout its lifecycle to ensure responsible and accountable data management. This information is based on the source document. from the official TriNetX website (https://trinetx.com/trust-privacy/).

**B. Data Security and De-Identification**

TriNetX is recognized as an ISO/IEC 27001:2013 certified entity, demonstrating its commitment to maintaining a robust Information Security Management System (ISMS). Data security measures emphasize a risk-based methodology to safeguard information assets. To ensure patient privacy and comply with regulations:

- All data provided to TriNetX by the HCOs undergoes pseudonymization and/or de-identification.
- TriNetX employs the Expert Determination method to guarantee information is adequately de-identified in accordance with the HIPAA Privacy Rule.
- The system adheres to standards set by the Brazilian General Data Protection Law (LGPD) and has established protocols aligning with the European Union's (EU) criteria for extraterritorial data transfer.
- A dedicated Data Trust team evaluates data throughout its lifecycle to ensure responsible and accountable data management.
